# Impact of temperature change from admission to day one on neonatal mortality in a low-resource setting

**DOI:** 10.1186/s12884-020-03343-7

**Published:** 2020-10-23

**Authors:** Francesco Cavallin, Serena Calgaro, Valentina Brugnolaro, Amir Hussein Abubacar Seni, Arlindo Rosario Muhelo, Liviana Da Dalt, Giovanni Putoto, Daniele Trevisanuto

**Affiliations:** 1 Independent statistician, Solagna, Italy; 2 Doctors with Africa CUAMM, Padova, Italy; 3 Department of Women and Children Health, University of Padova, Via Giustiniani, 3, 35128 Padova, Italy; 4 Central Hospital of Beira, Beira, Mozambique

**Keywords:** Birth, Hypothermia, Low-resource setting, Mortality, Temperature

## Abstract

**Background:**

Thermal control after birth is an essential part of neonatal care. However, the relationship between neonatal temperature at and after admission is unknown. This study aimed to evaluate the change between neonatal temperature at admission and at day 1, and its impact on mortality.

**Methods:**

Retrospective observational study at the Beira Central Hospital, Mozambique. Axillary temperatures were recorded at admission and at day 1 in 1,226 neonates who were admitted to the Special Care Unit between January 1 and December 31, 2017. The relationship between mortality rate and temperature change was evaluated with a matrix plot and a forest plot (obtained from a logistic regression model as odds ratios with 95% confidence intervals).

**Results:**

Normothermia was found in 415 neonates (33.8%) at admission and in 638 neonates (52.0%) at day 1. Mortality rate was highest in (i) neonates who remained in severe/moderate hypothermia (74%), (ii) neonates who rewarmed from hypothermia (40–55%), and (iii) neonates who chilled to severe/moderate hypothermia (38–43%). Multivariable analysis confirmed that temperature change from admission to day 1 was an independent predictor of mortality (p < 0.0001).

**Conclusions:**

In a low-resource setting, one out of three neonates was found hypothermic at day 1 irrespectively of admission temperature. Relevant thermal deviations occurred in a high proportion of newborns with normothermia at admission. Being cold at admission and becoming cold or hyperthermic at day 1 were associated with increased likelihood of mortality. Appropriate actions to prevent both hypothermia and hyperthermia represent both a challenge and a priority during postnatal period.

## Background

Maintaining normothermia at birth remains a major challenge in neonatal care [1, 2]. Neonatal hypothermia contributes to mortality as comorbidity of preterm birth, asphyxia and sepsis [3], while neonatal hyperthermia is associated with brain injury and hemodynamic changes [4]. Although deviations from normothermia are common in both high- and low-resource settings, maintaining normothermia at birth is even more critical in low-resource settings, where limited availability of equipment, poor provider training and inadequate awareness of the problem impair the thermal care of the newborns [3, 5].

Neonatal temperature at admission plays an important role in newborn survival in both high- and low-resource settings [6]. Previous studies showed that both hypothermia and hyperthermia at admission are associated with increased likelihood of mortality [6]. Therefore, appropriate actions to prevent thermal losses immediately after birth are crucial.

Furthermore, neonatal temperature during the first days of life has also been recognized as an important prognostic factor [7], thus highlighting the importance of thermal care during an extended period after birth.

However, little is known about the relationship between neonatal temperature at and after admission. In other words, it is unclear whether normothermia at admission is followed by negligible thermal losses, or whether deviations from normothermia (i.e. hypothermia or hyperthermia) improve after admission.

This study aimed to evaluate the change between neonatal temperature at admission and at day 1, and its impact on mortality in a low-resource setting.

**Fig. 1:**
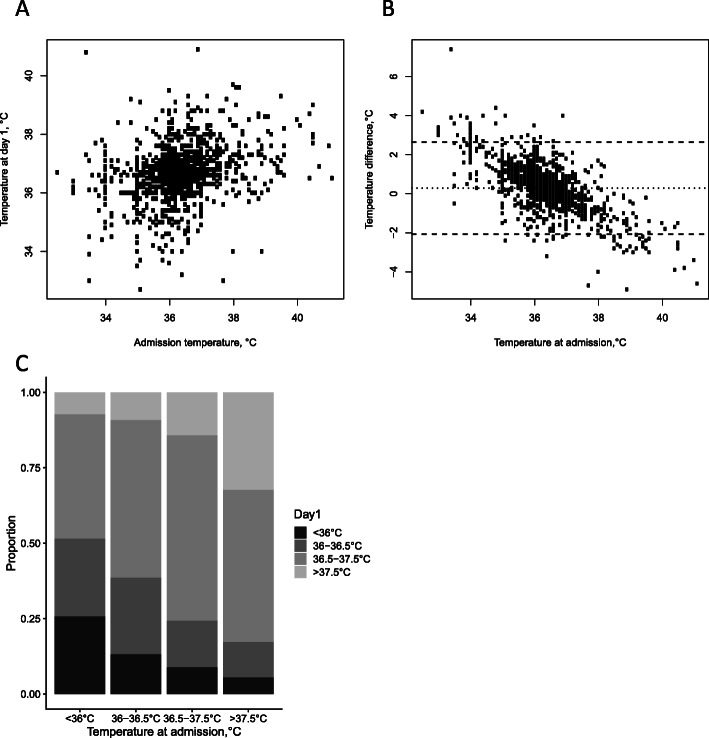
Neonatal temperatures at admission and day 1: scatterplot and boxplots (**a**), Bland-Altman-like plot (**b**), and change in temperature categories (**c**)

**Fig. 2:**
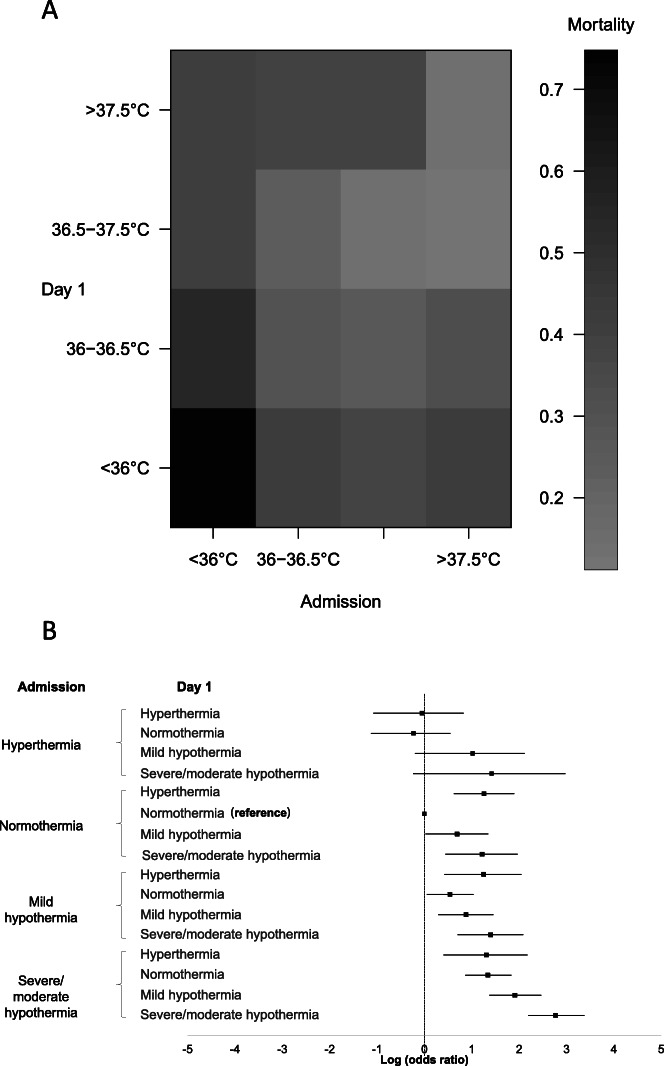
Mortality rate according to change in neonatal temperatures from admission to day 1: matrix plot (**a**) and forest plot (with log odds ratios and 95% confidence intervals with respect to neonates who remained in normothermia) (**b**)

## Methods

### Study design

This is a retrospective study about the effect of temperature at day 1 on neonatal mortality at the Special Care Unit (SCU) of the Beira Central Hospital (BCH) in Beira (Mozambique). The Clinical Board of BCH approved the study and waived the need for written informed consent given the retrospective nature of the study and the use of anonymized data from hospital records. BCH is located in the province of Sofala, Mozambique, and is the referral hospital for a geographical area that covers about 1.7 million people. About 5,000 deliveries and 2,100 admissions to the SCU occur every year at BCH.

### Patients

All neonates admitted to the SCU between January 1 and December 31, 2017 were evaluated for inclusion in the study. Neonates with available data on temperature at admission and at day 1 were included the study.

### Data collection

All data were retrieved from hospital records by hospital staff and were collected in an anonymized dataset. Data included birthplace, mode of delivery, gestational age, sex, birth weight, twin pregnancy, 5-minute Apgar score, diagnosis at admission, neonatal temperature at admission, and neonatal temperature at day 1. As the availability of laboratory and instrumental exams was limited, diagnosis at admission was based on clinical examination [8]. Neonatal axillary temperature was measured using an electronic thermometer. Severe/moderate hypothermia was defined as temperature < 36 °C, mild hypothermia as 36-36.4 °C, normal temperature as 36.5–37.5 °C and hyperthermia as > 37.5 °C [9].

### Statistical analysis

Continuous data were reported as median and interquartile range (IQR), while categorical data as number and percentage. Correlation between continuous data was evaluated using Spearman’s rank correlation coefficient (rho).

A Bland-Altman-like plot was used to graphically evaluate the distribution of the changes in neonatal temperature from admission to day 1 with respect to the temperature at admission.

The relationship between mortality rate and temperature change was evaluated with a matrix plot and a forest plot (obtained from a logistic regression model as odds ratios with 95% confidence intervals).

Multivariable analysis of mortality was performed with a logistic regression model including temperature change and a set of clinically relevant factors (jaundice/hyperbilirubinemia, fever, trauma, wet lung, asphyxia/HIE, sepsis/seizures, prematurity/LBW, congenital malformations, place and mode of delivery, HIV, sex, twin birth, maternal age, and number of previous gestations). The 5-minute Apgar score was not included because it was unavailable in neonates born at home. Gestational age and birth weight were included in the multivariable analysis as the clinically relevant category “prematurity/LBW”. Since all neonates born from caesarean sections were inborns, birthplace and mode of delivery were collapsed in one variable with four categories (“caesarean inborn”, “vaginal inborn”, “vaginal outborn” and “vaginal homebirth”). Model selection was performed by AIC reduction. In the final model, odds ratios (with 95% confidence intervals) were calculated to describe the effects of the independent variables on mortality. Model performance was evaluated with internal validation (c-index) and calibration (calibration-in-the-large and calibration slope) using bootstrap methods (re-sampling with replacement to create 1,000 samples of the same size as the original) [10].

All test were 2-sided and a p-value less than 0.05 was considered statistically significant. Statistical analysis was performed using R 3.5 (R Foundation for Statistical Computing, Vienna, Austria) [11].

## Results

A total of 1,344 neonates admitted to SCU from 1st January 2017 to 31st December 2017 were included in the analysis. Fifteen neonates were discharged alive within 24 hours after admission and 60 died within 24 hours after admission, while the information was not available in 8 neonates. Among the remaining 1261 neonates, neonatal temperature at day 1 was available in 1,226 neonates (97%) who were included in the analysis. Patient characteristics are reported in Table 1.

**Table 1:** Patient characteristics

	Temperature at admission				
	Any temperature	Severe or moderate hypothermia	Mild hypothermia	Normothermia	Hyperthermia
No. of subjects	1,226	345	339	415	127
*Neonates:*					
Gestational age, weeks ^ab^	37 (34-39)	36 (32-38)	37 (35-39)	37 (35-39)	39 (37-40)
Sex male:female ^c^	686:539	192:152	190:149	229:186	75:52
Birth weight, grams ^ac^	2450 (1700-3050)	1900 (1440-2725)	2280 (1700-3000)	2700 (1900-3100)	3000 (2500-3400)
Twin	237 (19.3)	86 (24.9)	72 (21.2)	70 (16.9)	9 (7.1)
5-minute Apgar score ^ad^	8 (6-9)	7 (5-8)	8 (6-9)	8 (7-9)	9 (8-10)
Mode of delivery: ^e^					
Vaginal	908 (74.5)	264 (77.2)	234 (69.0)	305 (74.2)	105 (83.3)
Caesarean	310 (25.5)	78 (22.8)	105 (31.0)	106 (25.8)	21 (16.7)
Birthplace					
Inborn	688 (56.1)	185 (53.6)	220 (64.9)	242 (58.3)	41 (32.2)
Outborn	451 (36.8)	121 (35.1)	104 (30.7)	147 (35.4)	79 (62.2)
Homebirth	87 (7.1)	39 (11.3)	15 (4.4)	26 (6.3)	7 (5.5)
Total time from birth to admission, minutes ^af^	61 (38-187)	55 (37-135)	52 (35-119)	70 (40-240)	868 (134-1580)
Diagnosis at admission:					
Asphyxia/HIE	324 (26.4)	102 (29.6)	101 (29.8)	101 (24.3)	20 (15.7)
Prematurity/LBW	409 (33.4)	169 (49.0)	122 (36.0)	105 (25.3)	13 (10.2)
Sepsis	32 (2.6)	1 (0.3)	7 (1.0)	4 (1.0)	20 (15.7)
Fever	50 (4.1)	3 (0.9)	0 (0.0)	8 (1.9)	39 (30.7)
CMs	48 (3.9)	8 (2.3)	16 (4.7)	19 (4.6)	5 (3.9)
Wet lung	118 (9.6)	25 (7.2)	30 (8.8)	58 (14.0)	5 (3.9)
Trauma	9 (0.7)	2 (0.6)	2 (0.6)	5 (1.2)	0 (0.0)
Seizures	12 (1.0)	1 (0.3)	2 (0.6)	5 (1.2)	4 (3.1)
Jaundice/ hyperbilirubinemia	22 (1.8)	1 (0.3)	6 (1.8)	11 (2.6)	4 (3.1)
Other diagnoses	202 (16.5)	33 (9.6)	53 (15.6)	99 (23.8)	17 (13.4)
*Mothers:*					
Maternal age, years ^ag^	23 (19-28)	23 (19-28)	23 (19-28)	23 (19-28)	23 (20-30)
Number of previous gestations ^ah^	2 (1-4)	2 (1-4)	2 (1-4)	2 (1-4)	2 (1-3)
HIV positive mother ^g^	300 (25.5)	100 (30.2)	83 (25.5)	88 (22.0)	29 (24.0)

Data expressed as No. (%) or ^a^ median (IQR)

CMs: Congenital malformations. *HIE* Hypoxic ischemic encephalopathy, *LBW* Low birth weight

Data not available in ^b^68, ^c^1, ^d^122, ^e^8, ^f^526 and ^g^49 subjects

Neonatal temperatures at admission and day 1 are shown in Fig. 1. Median neonatal temperature at admission was 36.3 °C (IQR 35.9–36.9 °C; min 32.5 °C, max 41.1 °C), with severe/moderate hypothermia reported in 345 neonates (28.1%), mild hypothermia in 339 (27.7%), normal temperature in 415 (33.8%) and hyperthermia in 127 (10.4%).

Median neonatal temperature at day 1 was 36.7 °C (IQR 36.2–37.1 °C; min 32.7 °C, max 40.9 °C), with severe/moderate hypothermia reported in 178 neonates (14.5%), mild hypothermia in 254 (20.7%), normal temperature in 638 (52.0%) and hyperthermia in 156 (12.8%).

Neonatal temperatures at admission and at day 1 were correlated (Spearman’s rho 0.31, *p *< 0.0001; Fig. 1A). Mean temperature change from admission to day 1 was 0.3 °C (with 95% of data between − 2.1 °C and 2.6 °C; Fig. 1B), and it was inversely correlated with temperature at admission (Spearman’s rho − 0.20, *p* < 0.0001).

The association between mortality rates and temperatures at admission and day 1 are shown in Fig. 2. Mortality rate was the lowest in neonates who remained in normothermia (15%) and in those who changed from hyperthermia at admission to normothermia/hyperthermia at day 1 (12–15%). On the other hand, mortality rate was the highest in (i) neonates who remained in severe/moderate hypothermia (74%), (ii) neonates who rewarmed from hypothermia (40–55%), and (iii) neonates who chilled to severe/moderate hypothermia (38–43%) (Fig. 2A; Table 2).

**Table 2:** Neonatal temperatures ad admission and at day 1: number of neonates and deaths (in brackets) for each category

		Neonatal temperature at admission			
		> 37.5 °C	36.5–37.5 °C	36-36.5 °C	< 36 °C
Neonatal temperature at day 1	> 37.5 °C	41 (6 deaths)	59 (23 deaths)	31 (12 deaths)	25 (10 deaths)
	36.5–37.5 °C	64 (8 deaths)	255 (39 deaths)	177 (42 deaths)	142 (58 deaths)
	36-36.5 °C	15 (5 deaths)	64 (17 deaths)	86 (26 deaths)	89 (49 deaths)
	< 36 °C	7 (3 deaths)	37 (14 deaths)	45 19 (deaths)	89 (66 deaths)

Unadjusted analysis of mortality showed that hypothermia at admission was a risk factor of mortality regardless of temperature at day 1 (Fig. 2B), while neonates admitted in normothermia had increased risk of mortality in case of both hypothermia and hyperthermia at day 1 (Fig. 2B). On the other hand, changing from hyperthermia at admission to hypothermia at day 1 had an inconclusive effect of mortality due to the small sample size (22 neonates).

At multivariable analysis of mortality, temperature change from admission to day 1, diagnosis, delivery, sex and HIV were included in the final model, while maternal age, number of previous deliveries and twin pregnancy were excluded (AIC reduction from 1169 to 1121). Temperature change from admission to day 1 (*p* < 0.0001), diagnosis (*p* < 0.0001), delivery (*p*< 0.0001) and HIV (*p* = 0.02) were associated with mortality, while sex (p = 0.13) did not. Odds ratio with 95% confidence intervals are reported in Table 3. Internal validation and calibration via bootstrapping showed good validation (c-index 0.78) and calibration (calibration-in-the-large − 0.0775 and calibration slope 0.8808).

**Table 3:** Multivariable analysis of mortality

Variable	*p*-value	Categories		Odds ratio (95% confidence interval)
Temperature change from admission to day 1	< 0.0001	Temperature at admission	Temperature at day 1	-
		Hyperthermia	Hyperthermia Normothermia Mild hypothermia Severe/moderate hypothermia	1.08 (0.33 to 3.03) 1.00 (0.39 to 2.38) 3.12 (0.84 to 10.56) 4.13 (0.65 to 23.63)
		Normothermia	Hyperthermia Normothermia Mild hypothermia Severe/moderate hypothermia	4.24 (2.11 to 8.49) *Reference* 2.48 (1.18 to 5.09) 3.59 (1.56 to 8.14)
		Mild hypothermia	Hyperthermia Normothermia Mild hypothermia Severe/moderate hypothermia	3.98 (1.64 to 9.45) 1.65 (0.96 to 2.83) 2.17 (1.15 to 4.10) 3.94 (1.81 to 8.54)
		Severe/moderate hypothermia	Hyperthermia Normothermia Mild hypothermia Severe/moderate hypothermia	3.45 (1.29 to 8.96) 3.46 (2.03 to 5.98) 6.28 (3.48 to 11.51) 11.24 (6.00 to 21.79)
Diagnosis at admission	< 0.0001	Fever, trauma, jaundice/hyperbilirubinemia, other Wet lung Asphyxia/HIE Sepsis/seizures Prematurity/LBW Congenital malformations		*Reference* 2.66 (1.37 to 5.24) 4.40 (2.64 to 7.59) 5.45 (2.27 to 12.89) 7.07 (4.31 to 12.06) 10.77 (4.82 to 24.57)
Sex	0.13	Female Male		*Reference* 1.26 (0.94 to 1.69)
Delivery	< 0.0001	Vaginal inborn Vaginal outborn Cesarean inborn Vaginal homebirth		*Reference* 1.35 (0.95 to 1.92) 0.59 (0.40 to 0.87) 2.18 (1.20 to 4.03)
HIV	0.02	No Yes		*Reference* 1.47 (1.07 to 2.02)

## Discussion

Our findings highlighted that one out of three neonates was found hypothermic at day 1 irrespectively of admission temperature. Both being cold at admission and becoming cold at day 1 were associated with increased likelihood of mortality.

To our knowledge, this is the first study investigating the relationship between temperatures at admission and at day 1, and assessing the impact of temperature change on mortality risk across a full range of neonatal temperatures in a low-resource setting. The importance of neonatal temperature at admission is well acknowledged, with deviations from normothermia being associated with adverse neonatal outcomes [6]. Available information clearly shows an association between hypothermia and mortality, but does not distinguish whether a causal relationship with mortality exists or the temperature is only an indicator of mortality risk [12]. While the mechanisms associated with the increased mortality are unclear, it has been hypothesized that alterations of normal metabolic functions during hypothermia may lead to clinical complications such as pulmonary hypertension, hypoglycemia, hyperkalemia, impaired fluid balance, or an accumulation of toxic metabolic by-products that may not be compatible with life [13]. On the other hand, hyperthermia has been associated with life-threatening events such as neonatal seizures, hypotonia, and apnea [1, 14].

It is reasonable to think that the threat of deviations from normothermia does not expire after admission, thus requiring further attention during neonatal care. A previous review suggested that neonatal temperature during the first days of life can represent an important contributor to neonatal mortality in low-resource settings [7]. However, there is lack of information about the relationship between neonatal temperature at and after admission, as well as the prognostic role of temperature variation after admission. The present study adds information on this aspect, and showed that a non-negligible proportion of neonates (one out of three) was found hypothermic at day 1 irrespectively of admission temperature. In addition, normothermia at admission was followed by relevant thermal deviations leading to four out of ten neonates shifting to hypothermia or hyperthermia at day 1. Our findings indicated that both being cold at admission and becoming cold at day 1 were risk factors for mortality. In addition, becoming hyperthermic at day 1 was also associated with increased likelihood of mortality.

The study has some limitations that should be considered when reading the results. First, it is a single-center retrospective study, thus the generalizability of the findings may be limited to similar settings. Second, diagnosis at admission was based on clinical examination due to limited availability of laboratory and instrumental exams.

Within its limitations, our findings provide useful information to clinicians and stakeholders about the importance of thermal control in the postnatal period. While the current approaches in preventing thermal deviations from normothermia usually focus on the period immediately after birth, more prolonged efforts are needed in the following postnatal period, since there is a non-negligible likelihood of hypothermia at day 1 irrespectively of admission temperature. Normothermia at admission may reasonably lead health care providers to consider the newborn at low risk of adverse outcomes. However, relevant thermal deviations are likely to occur in a high proportion of newborns with normothermia at admission, thus requiring more attention on thermal control of these subjects. Appropriate actions to prevent both hypothermia and hyperthermia during postnatal period are crucial, since being cold at admission and becoming cold or hyperthermic at day 1 were associated with increased likelihood of mortality.

Implementation of practices such as adequate room temperature, skin-to-skin contact, use of heat sources and continuous monitoring of neonatal temperature should be promoted especially in low-resource settings, where maintaining normothermia is hampered by several barriers [3, 5, 15].

## Conclusions

In a low-resource setting, one out of three neonates was found hypothermic at day 1 irrespectively of admission temperature. Relevant thermal deviations occurred in a high proportion of newborns with normothermia at admission. Being cold at admission and becoming cold or hyperthermic at day 1 were associated with increased likelihood of mortality. Appropriate actions to prevent both hypothermia and hyperthermia represent both a challenge and a priority during postnatal period.

## Data Availability

The datasets used and analyzed during the current study can be available from the corresponding author on reasonable request.

## Abbreviations

BCH: Beira Central Hospital
SCU: Special Care Unit
NICU: Neonatal intensive care unit
WHO: World Health Organization
